# Giant Saphenous Venous Graft Aneurysm with Compression of the Pulmonary Artery: A Rare Cause of Heart Failure

**DOI:** 10.1155/2015/854296

**Published:** 2015-11-16

**Authors:** Jagadeesh K. Kalavakunta, Yashwant Agrawal, Alicia Williams, Jerry W. Pratt, Frank Saltiel

**Affiliations:** ^1^Department of Cardiology and Cardiothoracic Surgery, Michigan State University/Borgess Medical Center, Kalamazoo, MI 49048, USA; ^2^Department of Internal Medicine-Pediatrics, Western Michigan University, Homer Stryker School of Medicine, Kalamazoo, MI 49048, USA

## Abstract

We report a case of a 74-year-old man who presented with dyspnea on exertion and pedal edema. He had five-vessel coronary artery bypass graft (CABG) surgery twenty-six years ago and redo three-vessel CABG done thirteen years later. Computed tomographic angiography (CTA) of the heart and coronary vessels demonstrated a giant aneurysm arising from the saphenous venous graft (SVG) to the first obtuse marginal of the left circumflex artery compressing the pulmonary artery (PA). He underwent coronary angiography, confirming the CTA findings. Surgical and percutaneous interventions were offered, but the patient opted for conservative management due to the high risk of morbidity and mortality.

## 1. Case Report

**Figure 1:**
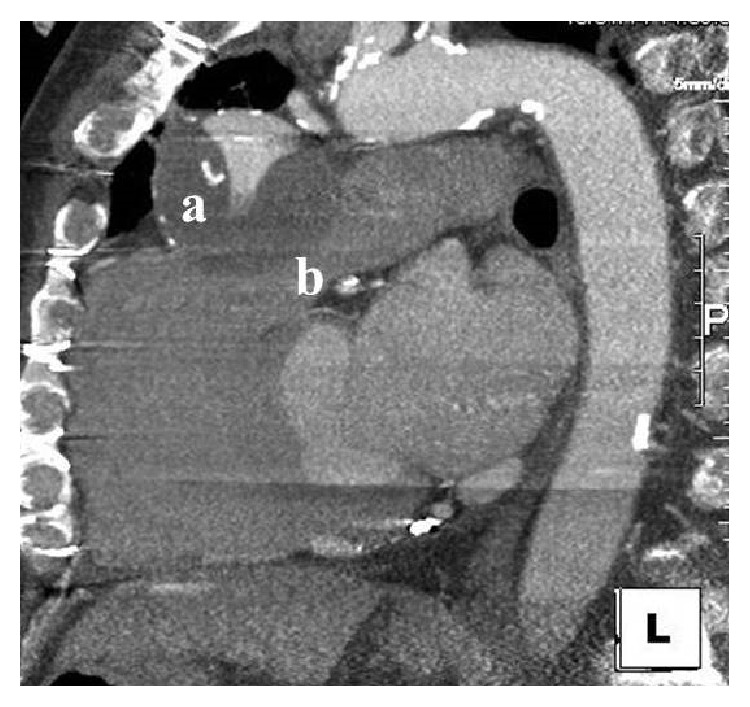
Coronary CT angiogram showing giant saphenous venous graft aneurysm (a) compressing the pulmonary artery (b).

**Figure 2:**
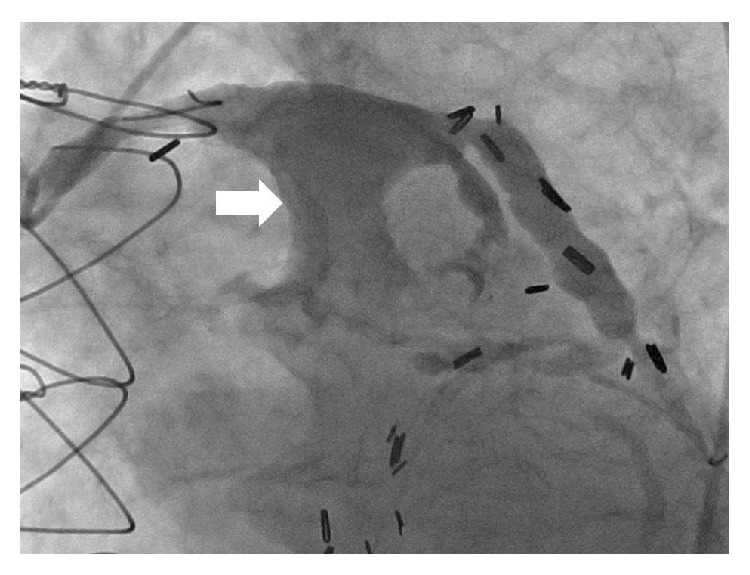
Saphenous venous graft angiography showing giant aneurysm (arrow) with organized thrombus.

A 74-year-old man presented to the clinic with lower extremity edema and exertional dyspnea for several weeks. Past medical history was significant for five-vessel aortocoronary artery bypass graft surgery (CABG) done in 1989 and redo three-vessel CABG in 2002. He had multiple comorbidities with 37-pack-year history of smoking. Physical examination was significant for grade II/VI systolic murmur at second left intercostal space, mild pitting edema, and jugular venous distention. Initial labs were unremarkable. Electrocardiogram revealed old anterior infarct changes. Transthoracic echocardiogram revealed severe pulmonary artery stenosis with mean gradient of 44 mmHg and peak gradient of 76 mmHg secondary to extrinsic compression of pulmonary artery by a large extrinsic mass. Right ventricular hypertrophy along with reduction of systolic function was also noted. Left ventricular ejection fraction was also reduced (30–35%) with anterior wall akinesis. Computed tomography angiography (CTA) of the heart and coronary vessels revealed a 6.0 × 5.8 cm aneurysm (Figure 1) involving the first obtuse marginal SVG, which was compressing the pulmonary artery. Coronary angiography was performed, which revealed 100% occlusion of all the three native vessels. SVG angiography revealed a 7 × 7 cm giant aneurysmal dilatation in the SVG to the first obtuse marginal of the left circumflex artery, which appeared to contain a large organized thrombus (Figure 2).

Cardiothoracic surgery service was consulted and they proposed excision of the aneurysmal vein graft and 2/3 vessel coronary artery bypass grafting using a radial artery and/or right internal mammary artery. Given his extensive comorbidities, previous cardiac surgeries, and significantly reduced left ventricular function, surgical therapy was felt to carry significantly increased risk of morbidity and mortality. Percutaneous intervention was also discussed, where exclusion of the aneurysm by insertion of a covered stent in the SVG across the neck of the aneurysm as well as possible stent implantation of the pulmonary artery would be performed. The aneurysm was followed up by repeat CTA scan showing an increase to 6.8 × 6.5 cm, but the patient opted for continued follow-up by CTA every 6 months.

## 2. Discussion

SVG aneurysm is a rare complication of CABG with reported incidence of 0.07% [1]. Atherosclerotic degenerative changes are the main cause of SVG aneurysm formation. Approximately one-third of the cases that undergo coronary angiography can have SVG aneurysms as a supplementary finding. SVG aneurysms can present with chest pain/angina (46.4%), dyspnea (12.9%), myocardial infarction (7.7%) [2], and rarely death as a result of fistula formation with other cardiac chambers, compression of various cardiac chambers and great vessels, aneurysm rupture, hemothorax, and cardiac tamponade.

Case reports of giant SVG aneurysm compressing pulmonary artery, right atrium, and right ventricle have been previously reported [2, 3]. Presentation from compression is generally of signs and symptoms from decompensated right failure. This patient presented to the clinic with early stages of right heart failure despite severe degree of pulmonary artery stenosis which is rare.

Treatment options available for the repair of giant SVG aneurysms lack specific guidelines. Surgical ligation of graft with aneurysmal excision and redo bypass grafting of native coronary artery is most typically performed [4]. Percutaneous approach has also been reported in a few cases [4, 5], though early intervention with either percutaneous or surgical approach prevents complications including embolization. Thorough evaluation by the multidisciplinary team is appropriate considering the complex nature of the lesion, rare incidence, and often-high risk involved in the procedure.

Despite its very rare nature, SVG aneurysm with severe pulmonary artery stenosis carries a high morbidity and mortality risk given the high likelihood of calamitous complications including the potential for sudden cardiac death. Since early recognition often allows for greater treatment options, physicians should maintain a high index of suspicion for this unusual graft complication in post-CABG patients who present with unexplained right sided congestive heart failure symptoms, or new radiographic and echocardiographic findings.
